# “Asking Too Much?”: Randomized N-of-1 Trial Exploring Patient Preferences and Measurement Reactivity to Frequent Use of Remote Multidimensional Pain Assessments in Children and Young People With Juvenile Idiopathic Arthritis

**DOI:** 10.2196/14503

**Published:** 2020-01-30

**Authors:** Rebecca Rachael Lee, Stephanie Shoop-Worrall, Amir Rashid, Wendy Thomson, Lis Cordingley

**Affiliations:** 1 National Institute for Health Research Manchester Musculoskeletal Biomedical Research Centre Central Manchester University Hospitals NHS Foundation Trust Manchester Academic Health Science Centre Manchester United Kingdom; 2 Centre for Epidemiology Versus Arthritis, Centre for Musculoskeletal Research Faculty of Biology, Medicine and Health The University of Manchester Manchester United Kingdom; 3 Centre for Health Informatics The University of Manchester Manchester United Kingdom; 4 Centre for Genetics and Genomics Versus Arthritis, Centre for Musculoskeletal Research Faculty of Biology, Medicine and Health The University of Manchester Manchester United Kingdom; 5 Division of Musculoskeletal and Dermatological Sciences Faculty of Biology, Medicine and Health The University of Manchester Manchester United Kingdom

**Keywords:** mHealth, pain, pain assessment, juvenile idiopathic arthritis, patient reported outcomes, pediatrics

## Abstract

**Background:**

Remote monitoring of pain using multidimensional mobile health (mHealth) assessment tools is increasingly being adopted in research and care. This assessment method is valuable because it is challenging to capture pain histories, particularly in children and young people in diseases where pain patterns can be complex, such as juvenile idiopathic arthritis (JIA). With the growth of mHealth measures and more frequent assessment, it is important to explore patient preferences for the timing and frequency of administration of such tools and consider whether certain administrative patterns can directly impact on children’s pain experiences.

**Objective:**

This study aimed to explore the feasibility and influence (in terms of objective and subjective measurement reactivity) of several time sampling strategies in remote multidimensional pain reporting.

**Methods:**

An N-of-1 trial was conducted in a subset of children and young people with JIA and their parents recruited to a UK cohort study. Children were allocated to 1 of 4 groups. Each group followed a different schedule of completion of MPT for 8 consecutive weeks. Each schedule included 2 blocks, each comprising 4 different randomized time sampling strategies, with each strategy occurring once within each 4-week block. Children completed MPT according to time sampling strategies: once-a-day, twice-a-day, once-a-week, and as-and-when pain was experienced. Adherence to each strategy was calculated. Participants completed the Patient-Reported Outcomes Measurement Information System Pain Interference Scale at the end of each week to explore objective reactivity. Differences in pain interference scores between time sampling strategies were assessed graphically and using Friedman tests. Children and young people and their parents took part in a semistructured interview about their preferences for different time sampling strategies and to explore subjective reactivity.

**Results:**

A total of 14 children and young people (aged 7-16 years) and their parents participated. Adherence to pain reporting was higher in less intense time sampling strategies (once-a-week=63% [15/24]) compared with more intense time sampling strategies (twice-a-day=37.8% [127/336]). There were no statistically significant differences in pain interference scores between sampling strategies. Qualitative findings from interviews suggested that children preferred once-a-day (6/14, 43%) and as-and-when pain reporting (6/14, 43%). Creating routine was one of the most important factors for successful reporting, while still ensuring that comprehensive information about recent pain was captured.

**Conclusions:**

Once-a-day pain reporting provides rich contextual information. Although patients were less adherent to this preferred sampling strategy, once-a-day reporting still provides more frequent assessment opportunities compared with other less intense or overburdensome schedules. Important issues for the design of studies and care incorporating momentary assessment techniques were identified. We demonstrate that patient reporting preferences are key to accommodate and are important where data capture quality is key. Our findings support frequent administration of such tools, using daily reporting methods where possible.

## Introduction

### Chronic Pain in Juvenile Idiopathic Arthritis

Chronic pain is defined as pain that is unpleasant and long lasting with sensory, emotional, cognitive, and social components [1,2]. In children and young people with juvenile idiopathic arthritis (JIA), pain is also unpredictable, which hugely contributes to the burden of living with this long-term disease [3-5]. Children and young people conceptualize their JIA in terms of pain experienced [6], suggesting that for some, this is the most salient feature of their illness. However, health care professionals in pediatric rheumatology sometimes neglect to assess pain because of a lack of time and tools available to do so. In addition, professionals may not perceive pain as a priority for their patients because the focus of consultations may be on disease activity and function rather than pain [7,8]. Pain conversations are difficult to have with patients. The use of assessment tools, which remotely and efficiently collect rich pain data for these patients, could help to overcome this clinical problem.

### Multidimensional Mobile Health Pain Assessment

In the general field of pediatric pain, multidimensional assessment using electronic mobile health (mHealth) apps is growing widely and quickly [9]. These tools offer several advantages to researchers, clinicians, and patients, compared with unidimensional, paper-based versions. They enable accurate documentation of complex pain data, reporting patterns and pain fluctuations through time- and date-stamped reports [10-12]; demonstrate better adherence and engagement; avoid recall bias through timely monitoring [13]; and allow for wireless data transport, which can be useful for remote management of symptoms [14].

Remote monitoring of pain for children and young people with JIA is valuable; however, there are some specific administrative challenges for this patient group that need to be further explored before these tools can be effectively implemented. Given that the nature of pain qualities can change daily (and within days) for children with JIA [15], pain assessment should be not only multidimensional but also regular and frequent. However, in diary studies of pediatric pain, there is no consensus on the frequency of reporting. Some studies have adhered to once-a-day reporting, and others have implemented 3-times-a-day sampling strategies [16,17]. Studies also often only collect data for short periods (eg, 1 or 2 weeks) [18]. To our knowledge, no research to date has explored patient preferences in the timing and frequency of ecological momentary assessments (EMAs). EMA is a technique used to assess an individual’s current experiences, as they occur in real time and real-world settings [19]. In most chronic pain studies using EMA, the time sampling strategies are based on researcher decisions, which have little rationale, and are limited in regard to what is acceptable and feasible for patients [20].

### Frequency of Administration

Decisions regarding the frequency of pain assessment administration rarely appear to be evidence based. This is a particular concern for health care professionals managing children and young people with JIA because they fear that more intense, regular pain assessment may lead to over-reporting or overexaggeration of pain or pain-related problems [7]. This phenomenon is called measurement reactivity, which is defined as a change in the variable being measured, because of the nature of the measurement method [21,22]. To our knowledge, this effect has not been explored in electronic multidimensional pediatric pain assessment, but now that real-time data collection techniques are becoming commonplace in pediatrics [18], it is important to ensure that these assessment methods are not detrimental to well-being. To this end, changes in the degree of interference caused by pain may provide a useful indicator of the impact of frequent pain assessment. Therapists involved in the care of those with JIA use pain interference rather than measures, such as simple pain intensity scales, to evaluate the outcome of their interventions [7,23]. This approach fits with a broader concept of pain as a *motivational state* rather than simply a somatic experience [24].

### Study Aim

This study aims to investigate patient preferences, feasibility, and influence of several time sampling strategies in remote multidimensional pain reporting. Feasibility studies are used to determine whether an intervention or method is appropriate for real-world use in particular patient groups [25]. In the context of this research study, feasibility referred to the practicability of different self-report schedules, and an N-of-1 trial design allowed for a comparison of these within the same individuals [26]. We aimed to explore which pain reporting patterns were nonburdensome for children and young people with a complex long-term disease and why from both patients’ and parents’ perspective (as little qualitative research into patients’ reasons for disengaging exists [20]). We also studied the effects of different pain reporting intensities to investigate whether there was any evidence of measurement reactivity in response to using these tools more or less frequently.

## Methods

### Study Design

This study was a randomized N-of-1 cross-over trial design, which explored the use of 4 different time sampling strategies: once-a-day, twice-a-day, once-a-week, and as-and-when children and young people had pain. These time sampling strategies were chosen based on earlier pilot work in which children and young people with JIA completed My Pain Tracker (MPT; an mHealth multidimensional pain assessment tool, discussed further in the section Materials and Measures) daily for 1 week and discussed how often they thought would be feasible to complete the tool. This study has been reported in accordance with the Consolidated Standards of Reporting Trials extension for reporting N-of-1 trials (Multimedia Appendix 1) [27].

### Sample and Recruitment

Children and young people and their parents were recruited from a UK prospective inception cohort study of childhood-onset inflammatory arthritis (the Childhood Arthritis Prospective Study [CAPS]). The CAPS study collects data longitudinally from individuals who were diagnosed with inflammatory arthritis (in at least one joint) present for at least 2 weeks under the age of 16 years and who attend 1 of 5 UK pediatric rheumatology centers [28]. Exclusion criteria are septic arthritis, hemarthrosis, and arthritis caused by malignancy/trauma or connective tissue disorders. CAPS began recruitment in 2001 and collects up to 10 years of data following initial presentation to pediatric rheumatology. New children continue to be recruited currently. Written informed consent was provided by proxies for all participants. Assent was also provided by children where appropriate. The study obtained ethical approval as an amendment to CAPS from the UK National Health Service (NHS): Health Research Authority (REC 02/8/104, IRAS 184042).

Eligible children and young people and their parents were identified and contacted by the lead researcher (RRL) to invite them to a participant recruitment event in August 2017 in Manchester, the United Kingdom. Suitability for inclusion in this substudy was based on age (between 5 and 16 years) and English speaking in addition to the CAPS inclusion criteria [28]. Invitation letters and information sheets were sent. Interested children and young people and their parents were encouraged to contact the study team to register and to confirm their attendance at the recruitment event where a presentation about MPTs development was given, consent/assent was taken, and instructional guidance packs were provided. Children and young people and their parents who were interested but unable to attend the event were enrolled in their own homes. These participants were visited by the lead researcher (RRL), who gave a brief demonstration of how MPT worked before enrollment (completion of consent/assent forms and provision of instructional guidance pack for the study).

### Randomization

**Figure 1:**
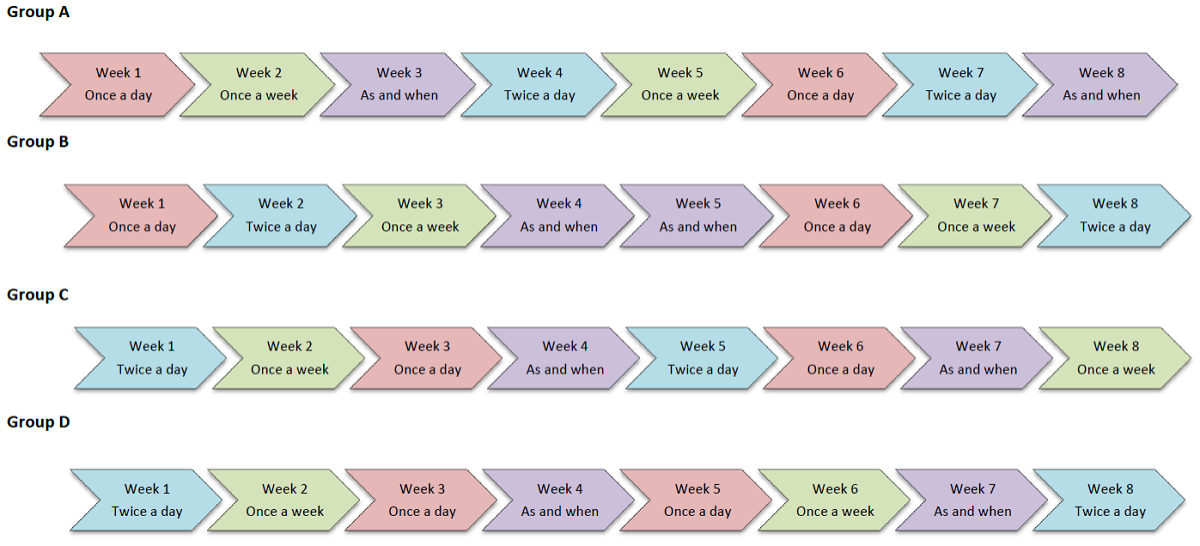
Randomised timing schedules.

Children and young people were randomized to 1 of 4 groups (group A, B, C, or D), as they presented to the study using a list of random numbers generated before recruitment by the lead researcher (RRL). Each group followed a different pain reporting schedule, which ran for 8 consecutive weeks, divided into 2 blocks of 4 weeks. Each pain reporting schedule included 4 different time sampling strategies (once-a-day, twice-a-day, once-a-week, and as-and-when the pain was experienced), which were randomized to repeat once within each of the 2 blocks. The randomized scheduling was created using block randomization and finalized before children and young people random allocation (see Figure 1). Children and young people were instructed not to complete MPT on the final day of each week (*washout* period) before cross over into another schedule to prevent carryover effects into the next schedule. As participants were not switching between interventions (or on any *active* treatments as part of the study design), only switching between administrative patterns, the anticipation of carryover and lingering effects was minimal. However, this 1-day washout time frame was chosen to balance the risk of any small carryover effects while ensuring that participants did not lose the momentum of using MPT at home. Information about reporting schedules and schedule changes was provided to participants in an instructional guidance pack, which also detailed who to contact if they had problems with MPT or the iPad. All randomization processes were created using Stata version 14.0.

### Setting

Data collection occurred in participants’ own homes throughout the North West of England.

### Materials and Measures

#### Remote Mobile Health Multidimensional Pain Assessment

**Figure 2:**
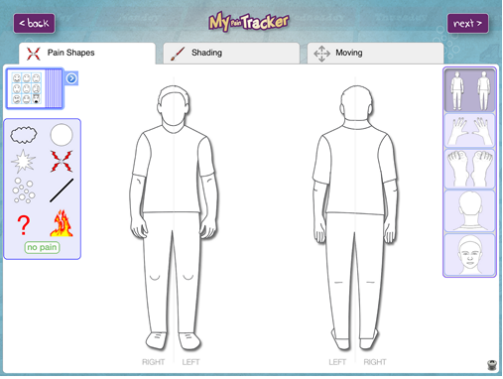
Main pain reporting page of My Pain Tracker.

Children and young people were provided with an iPad with MPT for the duration of the study. MPT is a multidimensional remote monitor of pain for children and young people with JIA. The tool’s development is long standing [29-33]. The software underpinning MPT and many of its graphical components first came from a developmentally appropriate computer-aided interview tool developed to facilitate children’s communication about somatic symptoms in a mental health context [30]. The tool was then adapted for use in acute postoperative pain, persistent pain, and, more recently, specifically in JIA [29,33,34]. MPT’s current format is an iPad app (version 1.6.5), which users manipulate to demonstrate pain experiences. Users of MPT are presented with a body manikin and are able to plot a number of different pain facets on the manikin to represent pain: location, symbols, labels/word descriptors, size (severity), throb rate (intensity), and emotion (see Figure 2 for the main user page of MPT). The app takes approximately 5 min to complete, but children and young people can complete it more or less quickly. After recording their pain using the app, an option is available on the main menu whereby users (including parents) can see their 9 most recent historical pain reports. Participants were contacted by the lead researcher at the end of each week to remind them to change to the new time sampling strategy for the following week.

#### Semistructured Interviews

Children and young people and their parents participated in a semistructured telephone interview following 8 weeks of data collection. The interview schedule consisted of questions about which administrative time sampling strategy children and young people and their parents liked the most and why and which was the most appropriate for long-term use of MPT. Children and young people and their parents were asked whether they observed or noted any changes in pain levels in response to the intensity of pain reporting (subjective pain reactivity at the end of the 8-week study period [35]). Telephone interviews lasted between 30 and 45 min and were audio-recorded and transcribed for analysis.

#### Pain Interference

Pain interference was assessed at the end of each week using the Patient-Reported Outcomes Measurement Information System (PROMIS) Pain Interference Scale-Short Form, which was either completed by children (those aged 8-16 years) or their parents (children younger than 8 years: the PROMIS Parent Proxy Pediatric Pain Interference Scale-Short Form) [36]. This measure was used to assess objective measurement reactivity (whether there were any actual changes in pain-related variables because of different measurement intensities [35]). Both the PROMIS pediatric and parent forms comprised 8 questions relating to the following aspects of pain interference: physical, psychological, and social functioning [36]. To score these measures, questions were scored on a scale of 0 to 4 (never to almost always) and summed for a final score out of 32 (higher scores represent greater pain interference). Scores could be completed in the presence of missing data if at least 50% of items had been answered. In these cases, overall scores were summed and converted to the same scale as if the total number of items had been completed. Previous work has shown that there is strong item agreement between parent- and patient-reported scores on PROMIS measures [37]. Completed questionnaires were returned in prepaid envelopes, as the study was ongoing.

### Data Analysis

#### Statistical Analyses

Adherence was calculated by examining the recorded number of MPT reports, and expected number of MPT entries completed within each time sampling strategy week (apart from as-and-when time sampling strategy, which had no expected number of entries). Quantitative analyses assessed any difference in pain interference scores when pain was recorded at different frequencies. This was primarily assessed graphically, with Friedman tests used to support conclusions drawn. Friedman tests assumed that pain interference did not systematically change week by week over the study duration, and there was no data autocorrelation by the participant. These assumptions were then assessed in secondary analyses.

The relationship between pain interference and time point (weeks in the study) was assessed using scatterplots and Spearman correlations. The reliability of pain interference scores over time within each time sampling strategy was tested using intraclass correlation coefficients (ICCs).

Previous work has suggested that within N-of-1 trials, simple comparisons of means tests outperform more complex methods, such as mixed effects models and meta-analyses, even in the presence of autocorrelation and carryover effects [38]. However, to confirm that autocorrelation at the participant level did not bias the conclusion drawn from the Friedman analysis, a multilevel linear regression tested differences in pain interference between the 4 time-sampling strategies. Strategies were compared against *once-a-day* strategy, and participant number was added as a random effect. All analyses were completed in accordance with an intention-to-treat principle using SPSS version 22 and Stata version 14.0.

#### Qualitative Analyses

Semistructured telephone interview data were analyzed through deductive semantic thematic analysis. Thematic analysis involves identification of meaningful patterns within data and generates rich, detailed accounts of participant’s perspectives [39]. Semantic analysis of data entails that the interpretation of data is largely rooted in the manifest content of participants’ accounts [40]. Recurring ideas and topics from children and young people and their parents were identified. These were organized into major and subthemes in NVivo 10. The themes were identified following a deductive approach, meaning predefined themes (about feasibility of time sampling strategies and perceptions of subjective reactive effects) formed the basis of the major themes. Narrative accounts of data phases were written, which were supported by children and young people and their parent quotations.

## Results

### Participant Characteristics

A total of 373 eligible CAPS participants were contacted to take part in the study, of which 20 potential participants registered. Of 373 participants, 8 children and their parents were able to attend a recruitment event, and a further 6 wished to be enrolled in the study at their homes. Moreover, 6 children and young people and their parents did not respond when contacted again directly by the researcher to organize a time to visit them.

Furthermore, 14 children and young people and their parents took part in the study. Children’s and young people’s demographics and disease characteristics are presented in Table 1. In addition, 2 participants experienced technical difficulties during the study, which meant that MPT data did not save to iPads. This highlighted issues with the technical feasibility of some of the MPT software and the tool itself (but this was not linked to the feasibility of the time sampling strategies tested). Therefore, MPT data from these 2 participants were excluded from the analysis of adherence. Their data were still included in other statistical analyses, as pain interference questionnaires were returned, and in the qualitative analyses, as semistructured interviews were still conducted. All data were collected between August 2017 and January 2018. The full age range for the participants included in the study was 7 to 16 years.

**Table 1:** Children and young people’s demographics and disease characteristics (N=14).

Characteristics		Values
Age at the study (years), median (IQR)		12.5 (10.0-14.0)
Disease duration at the time of the study (years), median (IQR)		4.3 (2.8-7.0)
Female, n (%)		9 (64)
**Subtypes of arthritis, n (%)**		
	Persistent oligoarthritis	6 (43)
	Extended oligoarthritis	1 (7)
	RF^a^-negative polyarthritis	5 (36)
	RF-positive polyarthritis	1 (7)
	Psoriatic arthritis	1 (7)

^a^RF: rheumatoid factor.

### Adherence

Overall adherence (n=12) for each time sampling strategy demonstrated that adherence to once-a-week reporting was highest (15/24, 63% possible reports) followed by once-a-day (85/168, 50.6% total possible reports) and twice-a-day (127/336, 37.8% possible reports) reporting. As-and-when reporting ranged from 0 to 7 reports during the 2 weeks this strategy occurred for participants.

### Measurement Effects of Pain Monitoring Frequency on Pain Interference

**Figure 3:**
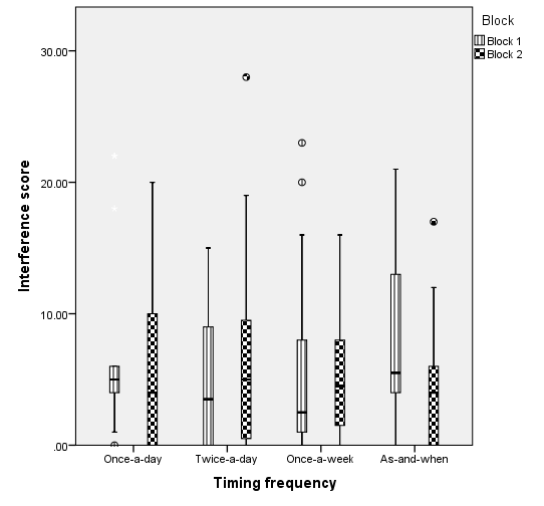
Boxplots of pain interference score distribution.

There were no systematic differences in pain interference between children and young people (n=14) in any of the different pain time sampling strategies using MPT (Figure 3; *P*=.77).

There was no correlation between week of participation and pain interference score (*r*=−0.04; *P*=.68). All time sampling strategies generated high test-retest reliability (all ICC over 0.6; see Table 2). When autocorrelation at the patient level was accounted for in a multilevel regression model, there were no significant differences in pain interference between time sampling strategies, compared with once-a-day strategy (compared with once-a-day strategy: once-a-week, *P*=.98; twice-a-day, *P*=.59; and as-and-when pain is experienced, *P*=.56).

**Table 2:** Median (IQR) of pain interference scores and correlation coefficients per time sampling strategy.

Time sampling strategy	Median (IQR) (maximum score of 32)			Intraclass correlation coefficient (significance)
	Block 1	Block 2	Blocks 1 and 2 combined	
Once-a-day	5.0 (3.0-12.0)	4.0 (0.0-12.5)	5.0 (3.0-10.0)	0.934
Twice-a-day	3.5 (0.0-9.0)	5.0 (0.0-13.0)	5.0 (0.0-9.0)	0.733
Once-a-week	2.5 (0.8-10.0)	4.5 (0.8-9.0)	5.0 (1.0-10.0)	0.630
As-and-when pain is experienced	5.5 (3.0-13.0)	4.0 (0.0-7.5)	5.0 (2.0-9.0)	0.898

### Overview of Qualitative Themes

A total of 2 major themes were deducted from the manifest content of interview data: theme 1: perceived advantages/disadvantages of each time sampling strategy and theme 2: perceived changes in pain experiences during the study (subjective measurement reactivity). Children and young people and their parent preferences for pain reporting strategies are presented in theme 1, and perceptions of subjective measurement reactivity are presented in theme 2, alongside narratives of findings (subthemes). Supporting interview quotations for each theme are presented in Multimedia Appendix 2.

### Theme 1: Perceived Advantages/Disadvantages of Each Time Sampling Strategy

An equal number of children and young people preferred once-a-day and as-and-when reporting (6/14, 43%). About 14% (2/14) of children and young people preferred twice-a-day reporting, with only one (1/14, 7%) participant reporting preference for once-a-week reporting. Within parents, 36% (5/13) reported that they most preferred once-a-day reporting, and slightly fewer preferred as-and-when strategy (4/13, 29%). Moreover, 14% (2/13) of parents preferred twice-a-day reporting, with one of these parents suggesting that an even more intensive time sampling strategy would have been appropriate (3 times a day). One parent (1/14, 7%) had no opinion on frequency of administration. Another parent preferred not to speak to the researcher about their preferences for reporting to encourage their child to be independently involved with the study by providing feedback about their use of the app on their own. This parent still provided consent and was present for the young person’s enrollment into the study.

### Subthemes

#### Once-a-Day

Reflecting upon, capturing, and storing comprehensive pain information appeared to be valuable to children and young people and meant they could *forget* about their pain until the next day. Children and young people found that they could capture pain variations between days, an advantage that could not be accommodated by less intensive time sampling strategies. Once-a-day reporting appeared to be the easiest to remember to complete because it became routine, whereby other regular activities (such as going to bed) provided a cue. For many of the children and young people, completion when they did not have pain became problematic, and completing the app daily became redundant in these cases. Children and young people who experienced pain daily suggested that MPT should include the option to report on how pain might have changed from morning to evening in this schedule. This seemed to show that although for some children and young people, once-a-day reporting was most feasible, capturing within-day variations in pain was still important.

Parents thought that the shorter recall period made the process of comprehending and reporting pain experiences much easier for children and young people. Some parents believed that longer time between reporting made it more difficult to think about what had happened in pain experiences since children had last reported. The preference for the once-a-day time sampling strategy seemed to be apparent for parents regardless of the level of pain, and as for some parents, it was also useful to know their child was not in pain.

#### Twice a Day

For some children and young people, twice-a-day reporting was most valuable because it captured within-day variations of pain (eg, the difference between morning and evening pain), which was not useful for those whose pain did not change. For other children and young people, it was difficult to space twice-a-day reporting out, so they did not complete 2 consecutive reports too closely together.

Many parents of children and young people expressed concern for how they would be able to manage this intensive reporting schedule when they were busy. Parents believed that because children were too busy to report, the child would rush the pain report and not show exactly how the pain felt. Owing to the intensity of this time sampling strategy, the information captured may have been less meaningful because less effort was put into completion when pain tracking was too frequent.

#### Once-a-Week

With this less intensive time sampling strategy, children and young people felt less pressure to fill MPT in, and they could *escape* thinking about pain. However, once-a-week reporting made it difficult to appreciate changes in pain from day to day. The amount of pain information condensed into the 1 weekly report could be problematic, particularly in those with multi-site pain. Children and young people would forget what data they had input during some weeks (how often they had reported and whether they still needed to report at all) or how their pain had been (what types of pain they had, how often, and where the pain was).

#### As-and-When Pain Is Experienced

The main advantage of as-and-when pain is experienced strategy was reporting flexibility. Some children and young people thought it was useful compared with reporting at times where they might not necessarily have experienced pain. Some children and young people liked that there was not a set amount of times that they had to report pain. The unpredictability of this timing schedule meant that the decision making of when to report was entirely upon the child, which for some was burdensome. For others, the flexibility of the time sampling strategy was problematic because they found it challenging to judge whether they had sufficient pain to report. Some children and young people believed that they should only report pain when it was bad. This was a disadvantage for some because it also meant that this time sampling strategy failed to capture information about when pain was better than usual.

Some children and young people did not like as-and-when reporting because this could be problematic when they were in school. They would forget about pain by the time they were home. For some, they would forget both about the pain they had experienced and to report because they were not prompted to do so. Some parents particularly preferred the as-and-when reporting because they felt that this kind of reporting alerted them to their child being in pain.

### Theme 2: Perceived Changes in Pain Experiences During the Study

The majority of children and young people (n=11) and their parents (n=11) did not perceive their pain to have been influenced by assessment frequency. For those who did report subjective reactivity (*children and young people*=3 and *parents*=2), it became apparent that there were 2 kinds of subjective measurement reactivity being referred to: cognitive/emotional and actual physical changes in the level of pain.

#### Subthemes

##### Cognitive/Emotional Reactivity

For a small number of children and young people (n=3), intensive pain reporting prompted them to think about pain more, which, in turn, made them more aware of it. These participants talked about how they would try to make a conscious effort to not let pain and thoughts about pain interfere with their day. Some children and young people in the study reported that focusing more on pain led them to notice smaller pains, which otherwise would have gone unnoticed or unreported. Children and young people talked about how they would only report pain that was worse than usual because some were used to a constant level of pain living with a chronic condition. Some parents believed that the bigger focus on pain affected their child’s mood and fatigue levels. Parents mentioned that mood and tiredness became worse because children were more aware of the pain (which parents believed their children would rather not think about) with higher frequency reporting. With this increase in the awareness of pain, parents talked about the difficulties of knowing whether to offer their child pain relief. These parents discussed how this difficult decision only seemed to arise when their child was more aware of how pain had felt in the more intense frequency time sampling schedules. It seemed that if children were not prompted to focus on pain for assessment, parents had more confidence in knowing that pain in and of itself was bothering their child.

##### Physical Pain Reactivity

One parent indicated that their child had experienced a reactive effect on pain severity in response to more intense reporting. In this particular interview, the child themselves did not perceive a change to have occurred. Pain was presumed to worsen by this parent because of the increased attention.

## Discussion

### Principal Findings

Although mHealth tools are increasingly being used to collect pain data in pediatric chronic pain [9], studies have failed to explore the impact of different time sampling strategies with patients and families [20]. It is important to explore this to develop administrative strategies, which balance reporting burden with the highest quality data collection techniques. Findings from this N-of-1 trial of different time sampling strategies suggest that statistically, there is no objective measurement reactivity with different pain reporting frequencies (in terms of pain interference). Qualitative findings suggest that children and young people and their parents have a preference for once-a-day and as-and-when reporting, but the disadvantages of as-and-when reporting (problematic flexibility and difficulties in remembering to report) far outweigh those cited for once-a-day. For some children and young people, there are perceived changes in emotion and fatigue in response to more intense pain reporting (subjective measurement reactivity). Children and young people demonstrate better adherence to less frequent time sampling strategies (once-a-week and once-a-day) in the short term. However, when reporting more flexibly (as-and-when pain is experienced strategy), some children and young people do not report pain at all, and for others, it can be problematic that *good* pain days are not captured.

Qualitative findings suggest that daily pain reporting is most feasible and preferred for children and young people with JIA, and quantitative data support that frequency of reporting has no impact on pain experience. Once-a-day strategy captured rich pain data, which children and young people valued and often looked back on in their pain report history (documented in MPT). This schedule enabled children and young people to explore patterns in pain (which would be useful for pain discussions with parents and/or health care professionals), and administration was perceived to be easily incorporated into routine. Although an equal proportion of children and young people preferred as-and-when reporting, there were more disadvantages cited for this time sampling strategy overall. For some, the flexibility of reporting was useful, but for others, this aspect of administration was burdensome because there were challenges associated with deciding when and how to report pain and which pains were significant. There were also difficulties associated with reporting pain experienced during school.

In addition to children’s and young people’s cited disadvantages, there are several methodological issues with event-based reporting, such as as-and-when pain is experienced (participant-initiated reports in response to pain occurrences [19]). In the interpretation of reports, it is impossible to know whether children did not have any pain or whether they did have pain but did not report it for whatever reason. This challenge would make it difficult to compare pain over time within individuals, which would be unfeasible in a clinical/home setting where there is remote data collection involved. Another problem concerns the data that are reported using as-and-when schedules. It is important to understand how and when children and young people define a painful event as occurring, and this would inevitably differ between participants [19]. As highlighted in the findings, some chose to only report unusually bad pain in as-and-when reporting. This schedule is problematic because the open interpretation of when it is necessary to report means that pain, which may be of interest to clinicians and researchers, is not captured.

In this study, patients’ preference was for daily pain reporting, whereas data entries were complete during once-a-week time sampling strategy, which may be an indication of the latter being a less demanding task. A strength of our work is that both subjective perspectives and objective indicators of completion were collected; however, the relative merits of each are important to consider. Lower adherence with a more frequent time sampling strategy still provides a more detailed picture (capturing daily variations) compared with complete but less intensive schedules. For example, a richer dataset is collected when a patient misses 3 of 7 daily reports (less adherent) compared with 1/1 weekly report (more adherent). From both research and health care professionals’ perspectives, a daily time sampling strategy with an incomplete dataset may appear more challenging to analyze, but it provides better contextual information about the pain experience overall. This advantage was also identified in the participant accounts.

### Comparisons With Prior Work

In the adult literature, studies using electronic pain diaries have found no evidence of measurement reactivity (objective or subjective) in participants’ data responses [35,41-43]. These papers, however, focused only on reactivity in terms of effects on pain intensity, rather than exploring the relationship between measurement artifacts and other pain-related variables (such as pain interference). Pain-related variables may offer richer contextual information, which is why we chose to explore it in this study. Measurement of pain interference is now recommended as a key outcome in clinical trials [44] and refers to measurement of the extent to which daily activities are interfered with or limited by pain. The assessment of interference is important, especially given that pain intensity does not necessarily correspond with lived experience [45].

It is difficult to disentangle changes in actual experiences (eg, natural fluctuations in pain interference) and whether these would occur regardless of any potential reactive effect, which is an inherent risk of bias in studies such as this [46]. There are ongoing discussions about how to better control for this bias, but to partly address this issue in this study design, the number of measurements was balanced, and the timing of measurement was randomized within individuals and between groups. Furthermore, participants completed *measurements* of reactivity separate to the intervention, which was being explored (MPT), as they were asked to complete an additional measure (PROMIS pain interference scale), which was administered at weekly time points.

In terms of the implications for clinical pain assessment for those with JIA, this study provides some reassurance for health care professionals that fears about pain focusing or pain overreporting may not be justified [7]. The children and young people and their parents in this study reported few differences in their pain experiences during more intense time sampling strategies. Our findings also have implications for pediatric pain research and clinical assessment of pain. Many pediatric pain studies use scales with time reference periods, which encompass substantial periods, such as 2 weeks or a month’s worth of pain [47]. This study showed that once-a-week reporting was one of the least appropriate time sampling strategies from patients’ and parents’ perspectives because children and young people struggle to condense large time frames of information into 1 singular report. This task is inevitably more difficult in even wider time reference periods.

### Strengths and Limitations

The substantial data collection period of this study should be considered a strength, as usually studies drawing on momentary assessment methodology in children and young people with chronic pain have narrow data collection periods (usually between 1, 2, or 3 weeks) [11,17,48]. A further strength of the study was the considerable sample size we recruited for the N-of-1 cross-over trials, which can be a burdensome study design for those involved. Very few N-of-1 trials have been conducted with children and where conducted in adults, it is not unusual to combine trial data from samples of less than 10 participants [26,49]. In N-of-1 trials, the number of data points per participant is considered to be more important than the total sample size [50].

A limitation of data collection is that participants were asked to verbally comment at the end of the study on whether they noticed changes in pain levels in response to the intensity of pain reporting. These subjective perceptions about measurement reactivity were thus based on their memory rather than objective assessment.

A limitation of this specific study design is that we did not explore the impact of random sampling protocols in children and young people (where randomly programmed iPad alerts would have prompted individuals when to report pain). This is the third main sampling type in real-time data collection techniques (in addition to time driven [based on preset schedules, such as once-a-day] and event-triggered [such as as-and-when schedule]). Exploring the feasibility of random scheduling would have allowed for a more complete picture of children’s experiences of using a wider range of momentary assessment techniques. A limit of the study findings is that N-of-1 trial findings are applicable to the patients the trial are conducted with and not for identifying population-level conclusions [51]. The findings of this intensive trial design possibly reflect the attitudes and impact of pain assessment frequency in some children and young people with JIA (we included participants of different ages and of different subtypes), but other patients may have different pain reporting needs that need to be investigated.

### Future Research

Future research should aim to explore the experience of perceived subjective measurement reactivity and the quality of these perceptions/changes in more detail. Several children and young people in our study reported some changes in mood and fatigue in their perceived response to more intense reporting schedules, which aligns with concerns that attention to pain leads to overexaggeration of symptoms expressed by health care professional’s perspectives [7]. Given these concerns, it would be interesting to explore health care professionals’ perspectives on these approaches. Further research should identify those patients for whom more frequent assessment of pain might be problematic and particularly emotionally or cognitively demanding. From this, we should develop phenotypes of these individuals to ensure appropriate pain data collection with minimal harm in pediatric pain studies and clinical care using these complex, multidimensional pain assessment tools.

### Conclusions

In conclusion, our study highlights that daily reporting of pain using mHealth multidimensional assessments is most feasible in terms of patient preference and adherence in long-term data collection with children and young people with JIA. There was no evidence to support that any timing schedule had an objective impact on pain interference, although there were some perceived changes in mood and fatigue in more intense reporting schedules for some participants. These findings are important for the development of administrative guidelines for remote pain monitoring tools, which accommodate momentary assessment techniques. Overall, our findings support the use of mHealth multidimensional pain assessment tools regularly and frequently to better capture pain patterns in children and young people with JIA.

## Appendix

Multimedia Appendix 1 Consolidated Standards of Reporting Trials extension for reporting N-of-1 trials checklist.

Multimedia Appendix 2 Supporting interview quotations for each identified theme.

## Abbreviations

CAPS: Childhood Arthritis Prospective Study
EMA: ecological momentary assessment
ICC: intraclass correlation coefficient
JIA: juvenile idiopathic arthritis
mHealth: mobile health
MPT: My Pain Tracker
NHS: National Health Service
PROMIS: Patient-Reported Outcomes Measurement Information System
