# Supplementary material for: “Asking Too Much?”: Randomized N-of-1 Trial Exploring Patient Preferences and Measurement Reactivity to Frequent Use of Remote Multidimensional Pain Assessments in Children and Young People With Juvenile Idiopathic Arthritis
Source: J Med Internet Res. 2020 Jan 30;22(1):e14503. doi: 10.2196/14503 (PMC7055814; doi:10.2196/14503)
Supplement: Multimedia Appendix 1 [file jmir_v22i1e14503_app1.docx]

| **Section topic** | **Number** | **Item** | **Page number** |
| --- | --- | --- | --- |
| **Title and abstract** | 1a | Identify as an “N-of-1 trial” in the title | Page 1, title page. |
|  | 1b | See specific CENT Guidance for abstracts: include a structured summary of trial design, methods (participants, interventions, objective, outcome, randomisation, blinding masking), results (numbers randomised, recruitment, numbers analysed, outcome, harms) and conclusions | Page 2. |
| **Introduction** | 2.a.1 | Scientific background and explanation of rational | Page 4, paragraph 1, 2 and 3. |
|  | 2.a.2 | Rationale for using N-of-1 approach | Page 4, paragraph 5. |
|  | 2b | Specific objectives or hypotheses | Page 4, paragraph 5. |
| **Methods** | 3a | Describe trial design, planned number of periods and duration of each period (including run-in and wash out if applicable) | Page 5, Randomisation section and Figure 1. |
|  | 3b | Important changes to methods after trial start | N/A. |
|  | 4a | Eligibility criteria for participants (diagnosis or disorder, diagnostic criteria, comorbid conditions and concurrent therapies) | Page 5, Participants and recruitment section. |
|  | 4b | Settings and locations where the data were collected | Page 6, Setting section. |
|  | 4c | Whether the trial represents a research study and if so, whether institutional ethics approval was obtained | Page 5, Participants and recruitment section. |
|  | 5 | The interventions for each period with sufficient details to allow replication, including how and when they were actually administered | Figure 1 shows administrative patterns followed per group. Page 6, Materials and measures provides details of the intervention used (My Pain Tracker). |
|  | 6.a.1 | Completely defined pre-specified primary and secondary outcome measures, including how and when they were assessed | Page 6, Materials and measures section. Provides details of My Pain Tracker, PROMIS Pain interference scale and semi-structured interviews. |
|  | 6.a.2 | Description and measurement properties (validity and reliability) of outcome assessment tools | Page 6, Materials and measures section. Provides details of My Pain Tracker, PROMIS Pain interference scale and semi-structured interviews. |
|  | 6b | Any changes to trial outcomes after the trial commenced, with reasons | N/A. |
|  | 7a | How sample size was determined | Not conducted as recruitment based upon participant availability. |
|  | 7b | When applicable, explanation of any interim analyses and stopping guidelines | N/A. |
|  | 8a | Whether the order of treatment periods was randomised, with rational, and method used to generate allocation sequences | Page 5, Randomisation section. |
|  | 8b | When applicable, type of randomisation; details of any restrictions (such as pairs, blocking) | Page 5, Randomisation section. |
|  | 8c | Full, intended sequence of periods | Figure 1. |
|  | 9 | Mechanism used to implement the random allocation sequence (such as sequentially numbered containers) describing any steps taken to conceal the sequence until interventions were assigned | Page 5, Randomisation section. |
|  | 10 | Who generated the random allocation sequence, who enrolled participants, and who assigned participants to interventions | Page 5, Participant and recruitment section, Randomisation section. |
|  | 11a | If done, who was blinded after assignment to interventions (for example, participants, care providers, those assessing outcomes) and how | Not possible to blind as participants had to be aware of how often they were reporting their pain. |
|  | 11b | If relevant, description of the similarity of interventions | Differences in administrative patterns described, no difference between the intervention itself (My Pain Tracker), just the method by which it was completed. |
|  | 12a | Methods used to summarize data and compare interventions for primary and secondary outcomes | Page 8, Data analysis section. |
|  | 12b | Methods for additional analyses, such as subgroup analyses and adjusted analyses (If done, methods of quantitative synthesis of individual trial data, including subgroup analyses, adjusted analyses, and how heterogeneity between participants was assessed) | N/A. |
|  | 12c | Statistical methods used to account for carryover effect, period effects and intra-subject correlation | No statistical methods used to account for carry-over effects, but participant ‘wash-out’ of previous schedule discussed on Page 5, Randomisation section. |
| **Results** | 13.a.1 | Number and sequence of periods completed, and any changes from original plan with reasons | Page 9, Adherence section. |
|  | 13.a.2 | The number of participants who were enrolled, assigned to interventions and analysed for the primary outcome | Page 8, Participant characteristics section. |
|  | 13c | Losses or exclusions of participants after treatment assignment, with reasons and period in which this occurred, if applicable | Page 8, Participant characteristics section. |
|  | 14a | Dates defining the periods of recruitment and follow-up | Page 9, Participant characteristics section. |
|  | 14b | Whether any periods were stopped early and/or whether trial was stopped early, with reasons | N/A |
|  | 15 | A table showing baseline demographic and clinical characteristics for each group | Page 9, Table 1, Participant characteristics section. |
|  | 16 | For each intervention, number of periods analysed. If quantitative synthesis was performed, number of trials for which data were synthesised | Page 9, Adherence section and Measurement effects of pain monitoring frequency on pain interference section. |
|  | 17.a.1 | For each primary and secondary outcome, results for each period; an accompanying figure displaying the trial data is recommended | Page 9, Measurement effects of pain monitoring frequency on pain interference section and Figure 3. |
|  | 17.a.2 | For each primary and secondary outcome, the estimated effect size and its precision (such as 95% CI) | N/A |
|  | 17b | For binary outcomes, presentation of both absolute and relative effect sizes is recommended | N/A |
|  | 18 | Results of any other analyses performed, including assessment of carryover effects, period effects, intra-subject correlation (if done, results of subgroup or sensitivity analyses) | Page 10, Overview of qualitative themes section. |
|  | 19 | All harms or unintended effects for each intervention | Page 11, Theme 1-percieved advantages/disadvantages of each time-sampling strategy section and Theme 2- Perceived changes in pain experiences during the study section. |
| **Discussion** | 20 | Trial limitations, addressing sources of potential bias, imprecision and if relevant multiplicity of analyses | Page 17, Strengths and limitations section. |
|  | 21 | Generalisability (external validity, applicability) of the trial findings | Page 17, Strengths and limitations section. |
|  | 22 | Interpretation consistent with results, balancing benefits and harms, and considering other relevant evidence | Page 16, Principle findings section and Comparisons with prior work section. |
| **Other information** | 23 | Registration number and name of trial registry | N/A |
|  | 24 | Where the full trial protocol can be accessed, if available | N/A |
|  | 25 | Sources of funding and other support (such as supply of drugs), role of funders | Page 18, Acknowledgements section. |
