# Supplementary material for: “Asking Too Much?”: Randomized N-of-1 Trial Exploring Patient Preferences and Measurement Reactivity to Frequent Use of Remote Multidimensional Pain Assessments in Children and Young People With Juvenile Idiopathic Arthritis
Source: J Med Internet Res. 2020 Jan 30;22(1):e14503. doi: 10.2196/14503 (PMC7055814; doi:10.2196/14503)
Supplement: Multimedia Appendix 2 [file jmir_v22i1e14503_app2.docx]

Multimedia Appendix 2: Themes, subthemes, and interview excerpts.

| Themes | Interview excerpts from children and young people | Interview excerpts from parents |
| --- | --- | --- |

| **Theme 1: perceived advantages/disadvantages of each time sampling strategy** | | | | | |
| --- | --- | --- | --- | --- | --- |
|  | **Subthemes** | | | | |
|  |  | **Once a day** | | | |
|  |  |  | Prompts children and young people to reflect on how pain has been | *Yes, it makes you think about your day and like, did you actually have pain then.* [Child 7] | —^a^ |
|  |  |  | Captures comprehensive pain information | *Once-a-day, ‘cause you’re getting lots of information...and it’s, like...and then you forget about it “til next time.”* [Child 6] | — |
|  |  |  | Captures pain variations between days | *Like if I did it, say once-a-week, I couldn’t really get down, like on Monday, I had a pain in my knee, but on Tuesday I didn’t. Doing it once-a-day, I could like...so my hand hurt on that day, my hip hurt on that day, kind of thing.* [Child 5] | — |
|  |  |  | Easy to remember | *So if you’re going through say a routine, every night, you’d do it.* [Child 5] | — |
|  |  |  | Incorporated into bedtime routine in particular | — | - *So, you know, we, sort of, tend to do it before she goes to bed ‘cause ...they’re just relaxing. We’re just calming down and, oh do your tracker. And then that’s it. The day is done.* [Parent of child 6] - *Before going to bed, she can settle down with it and just think about that.* [Parent of child 8] |
|  |  |  | Problematic for children and young people who do not have pain daily | - *If you didn’t have pain in them days...it kind of didn’t make sense to have to like keep putting like...I’ve not had a lot of pain.* [Child 9] - *If I didn’t have any pain at all, then I wouldn’t be able to fill it in.* [Child 13] | — |
|  |  |  | Still important to capture within-day variations of pain in once-a-day reporting | - *Like the once-a-day, I feel like maybe to improve it, there could be like a function that would be like, was it worse in the morning or in the afternoon for you?* [Child 3] - *Because when I was doing the once-a-day one, I was only doing it, like, before going to bed, so I couldn’t see how, like, painful my leg was in the morning.* [Child 4] | — |
|  |  |  | Short recall period better | — | *Everything’s kind of a bit fresh in your mind. You’ve only got that day’s worth of pain to put on. If it’s every couple of days, oh I had pain the other day, trying to remember and juggle that.* [Parent of child 6] |
|  |  |  | Useful for parents regardless of pain levels | — | *When someone’s not in much pain, I think once-a-day is useful.* [Parent of child 10] |
|  |  | **Twice a day** | | | |
|  |  |  | Captures within-day variations | *Because it wasn’t painful in the morning and then when I came home, it was more painful.* [Child 4] | — |
|  |  |  | Pain does not always change throughout the day | *Sometimes the pain stayed the same during the day so I felt like I was putting the same details in over and over again.* [Child 14] | — |
|  |  |  | Difficult to not report too closely together | *Whereas if it was the two one, I’d be like, well, if I did it at this time, I’d wait for a certain amount of time and I’d be like, wait, no, that’s not good*. [Child 5] | — |
|  |  |  | Parents think children and young people are too busy to report twice a day | — | *I know she gets pain first thing in the morning, but because we’re concentrating on getting to school, it’s the last thing on our list.* [Parent of child 8] |
|  |  |  | Children and young people would rush to complete pain reports | — | *Sometimes he’d rush and...I found not do it right ‘cause he felt like he has to do it...I think doing it before breakfast, sometimes he’d be like rushing out the door.* [Parent of child 12] |
|  |  |  | Children and young people are too busy | *Because I’m quite busy and sometimes you do forget.* [Child 2] | *—* |
|  |  | **Once a week** | | | |
|  |  |  | Less intense reporting and less pressure to report. | *I could just escape and I knew that was done for the week and then...because like it was constantly remembering them in the other times...like you know the pressure of just getting it done.* [Child 11] | — |
|  |  |  | Difficult to see changes in pain | *You couldn’t really see how painful it was everyday.* [Child 4] | — |
|  |  |  | Difficult to condense multisite pain information | - *There’s that much information to input, one day it will be my ankle and then another day it could be my ankle and my knee, then it could be my jaw. I might forget different types of pains.* [Child 8] - *So if I had pain more than once, I didn’t know when to fill it in, because if I had pain again, then I wouldn’t be able to put it on.* [Child 13] | — |
|  |  |  | Children and young people forgot how often they had reported that week | *You forgot when you filled it in and when you didn’t fill it in. Or then I kind of like kept forgetting that I had already filled it in.* [Child 14] | — |
|  |  |  | Children and young people forgot what type of pain they had had | *Say if you did it on Sunday, it was hard to remember what you felt on Monday.* [Child 9] | — |
|  |  |  | Children and young people forgot when they had pain | *Because if I could only do it once-a-week...you can forget a bit...Like I forgot when I have the pain.* [Child 2] | — |
|  |  | **As and when pain is experienced** | | |  |
|  |  |  | Flexibility in reporting important rather than set times | - *I preferred using as-and-when, rather than at set times.* [Child 9] - *Whenever I needed to, because it meant that you didn’t have to fill it in continuously.* [Child 14] | — |
|  |  |  | Difficult to know when to report pain | - *Sometimes, I didn’t know when to fill it in.* [Child 13] - *I don’t think I’ve had any pain but if I had to do it, I’ll be like, oh right, did I have pain...But I don’t...’cause if I have to think...but I don’t really think as hard as I would ‘cause I don’t actually have to do it.* [Child 6] | — |
|  |  |  | Only captures when pain is bad | *Because in my head the as-and-when one, I would just be like, oh, but I didn’t get any pain and then I couldn’t see that.* [Child 5] | — |
|  |  |  | Problematic when in school and children and young people forget how the pain has been | - *It’s just ’cause I might have pain somewhere, like at school, and you can’t have the iPad with you, so you can’t fill it in there. You would have to remember it and come home and do it then.* [Child 8] - *The iPads at home, if I have pain in school, I would then have to remember to put it into the log.* [Child 14] | — |
|  |  |  | Difficult to remember to report pain if not prompted to do so | *You forget sometimes...if you do it like once-a-day, it like makes you think about your day, did I have pain and stuff…because I didn’t take my iPad to school, sometimes I’d forget to put it in…some pain you just miss and you don’t really think about it.* [Child 7] | — |
|  |  |  | Useful to parents as then they know their child is in pain | — | *For us, the as-and-when is better, because then we can see when he’s actually having the pain, rather than just doing it every night.* [Parent of child 2] |
| **Theme 2: perceived changes in pain experiences during the study** | | | | | |
|  | **Subthemes** | | | | |
|  |  | No subjective pain reactivity | | - *Not particularly, it didn’t hurt more.* [Child 2] - *I found it better off my head, because it would like distract me from the pain.* [Child 4] | *He was able to have that time to focus on how it felt, to record it down. I don’t think it then made him feel any worse or better, or anything.* [Parent of child 13] |
|  |  | **Cognitive/emotional pain reactivity** | | | |
|  |  |  | More aware of the pain while reporting | *When you have to record it, it makes you more aware of your pain...I’d just be thinking about it for like, ten minutes and then I’d try and not let it affect what I was doing.* [Child 9] | — |
|  |  |  | Thinking about pain more makes children and young people notice smaller pains, which would have otherwise gone unreported | *So I think I kind of picked up on the smaller pain on them days, because I had to think about it more... I think when you think about it more, like my feet and my knees tend to hurt quite a bit.* [Child 14] | — |
|  |  |  | A reminder of how bad pain actually is | - *Like, because I obviously realised the pain, but I didn’t know it was that bad.* [Child 4] - *’Cause you want to forget about it through the day, ‘cause say if you do it in the morning, throughout the night ...when I think about it twice-a-day, I’m like...oh its quite a lot. I didn’t realise it.* [Child 6] | — |
|  |  |  | Increased awareness of pain affects mood and fatigue (from a parent’s perspective) | — | *Yeah I think she was more aware of where the pain was and how often she actually does get pain...it affects her mood and tiredness, she’d rather just not think about it.* [Parent of child 9] |
|  |  |  | Increased awareness of pain and worsening of mood and fatigue levels would prompt some parents to offer to provide additional pain relief to their child | — | *When they were more aware of how pain was and how often she actually does get pain, I was like, do you need more painkillers apart from the medication that you’re on.* [Parent of child 9] |
|  |  | **Physical pain reactivity** | | | |
|  |  |  | Children and young people with juvenile idiopathic arthritis used to a certain amount of pain. Increased awareness can worsen perceptions of the pain experienced. | — | *Yeah, I think there was a change in that, that she did actually think, oh yeah, well, this is actually hurting me, you know, whereas usually it wouldn’t. If you suffer with pain you get used to a certain amount of pain. So if you’re thinking about it more because you’re concentrating on it more, it does tend to worsen or you notice it more. You’re thinking ooh, well, I’ve got a pain there now.* [Parent of child 8] |

^a^—:
